# ‘Robot’ Hand Illusion under Delayed Visual Feedback: Relationship between the Senses of Ownership and Agency

**DOI:** 10.1371/journal.pone.0159619

**Published:** 2016-07-25

**Authors:** Mohamad Arif Fahmi Ismail, Sotaro Shimada

**Affiliations:** 1 Graduate School of Science and Technology, Meiji University, Kawasaki, Kanagawa, Japan; 2 School of Science and Technology, Meiji University, Kawasaki, Kanagawa, Japan; University of Bath, UNITED KINGDOM

## Abstract

The rubber hand illusion (RHI) is an illusion of the self-ownership of a rubber hand that is touched synchronously with one’s own hand. While the RHI relates to visual and tactile integration, we can also consider a similar illusion with visual and motor integration on a fake hand. We call this a “robot hand illusion” (RoHI), which relates to both the senses of ownership and agency. Here we investigate the effect of delayed visual feedback on the RoHI. Participants viewed a virtual computer graphic hand controlled by their hand movement recorded using a data glove device. We inserted delays of various lengths between the participant’s hand and the virtual hand movements (90–590 ms), and the RoHI effects for each delay condition were systematically tested using a questionnaire. The results showed that the participants felt significantly greater RoHI effects with temporal discrepancies of less than 190 ms compared with longer temporal discrepancies, both in the senses of ownership and agency. Additionally, participants felt significant, but weaker, RoHI effects with temporal discrepancies of 290–490 ms in the sense of agency, but not in the sense of ownership. The participants did not feel a RoHI with temporal discrepancies of 590 ms in either the senses of agency or ownership. Our results suggest that a time window of less than 200 ms is critical for multi-sensory integration processes constituting self-body image.

## Introduction

Self-body recognition happens naturally in our body, but the mechanism of self-body recognition is not yet clear. According to Gallagher, the basis of self-body recognition comprises two sensory components, the sense of ownership and the sense of agency [1–2]. The definition of the sense of ownership is the conscious awareness that the body belongs to us. The definition of the sense of agency refers to the sense of authorship of a given action. We can make a distinction between our own body and other people’s bodies because of these senses. However, the sense of ownership does not only occur toward our own body; there are a few cases in which we also feel a sense of ownership of other objects. An example of this case is the rubber hand illusion (RHI).

The RHI is a phenomenon whereby the participant perceives the simultaneous visual and tactile stimulation of an artificial hand (rubber hand) as their own tactile sensation, and sometimes attributes the rubber hand as being part of their own body [3–4]. Importantly, when the stimulus to the participant’s own hand and the rubber hand does not match in time, the RHI is greatly attenuated [5–6]. This phenomenon shows that self-body recognition can be established based on the temporal integrity of somatosensory information and visual information. If we stimulate the participant’s own hand without matching with the visual information, they will not feel the rubber hand as their own hand.

However, the time window for multisensory integration in self-body recognition is still unclear. Most studies have addressed this issue by comparing synchronous and asynchronous conditions, usually using a 500–1000 ms delay [5–6]. Our previous study investigated the effect of a delay of 100–600 ms with a 100-ms interval between the tactile stimulation of the rubber hand and that of the participant’s own hand, and the results showed that the attenuation in the degree of the RHI was observed for delays greater than 200–300 ms [7–8].

While the RHI is an illusion regarding visual and tactile integration, and hence concerns the sense of ownership, we can also consider a similar illusion regarding visual and motor integration on a fake hand. We call this the “robot hand illusion” (RoHI), and it is relevant to both the sense of ownership and the sense of agency. Previous research on the RoHI has not yet adequately addressed the effect of delay between these senses [9–12], although some studies have shown that participants feel the sense of ownership and the sense of agency of the robotic hand to be stronger in the synchronous than in the asynchronous condition. The present study more intensively investigated the sense of ownership and sense of agency in RoHI under delayed visual feedback of 90–590 ms.

## Methods

### Participants

Sixteen healthy students (12 male and 4 female; aged 21.0 ± 0.48 years, mean ± SD), who were right-handed and were not aware of the purpose of the study, were chosen for the experiment. All participants had normal or corrected-to-normal vision, and provided written informed consent. The experiment was approved by the ethics committee of the School of Science and Technology, Meiji University, and was according to the principles and guidelines of the Declaration of Helsinki.

### Apparatus

**Fig 1:**
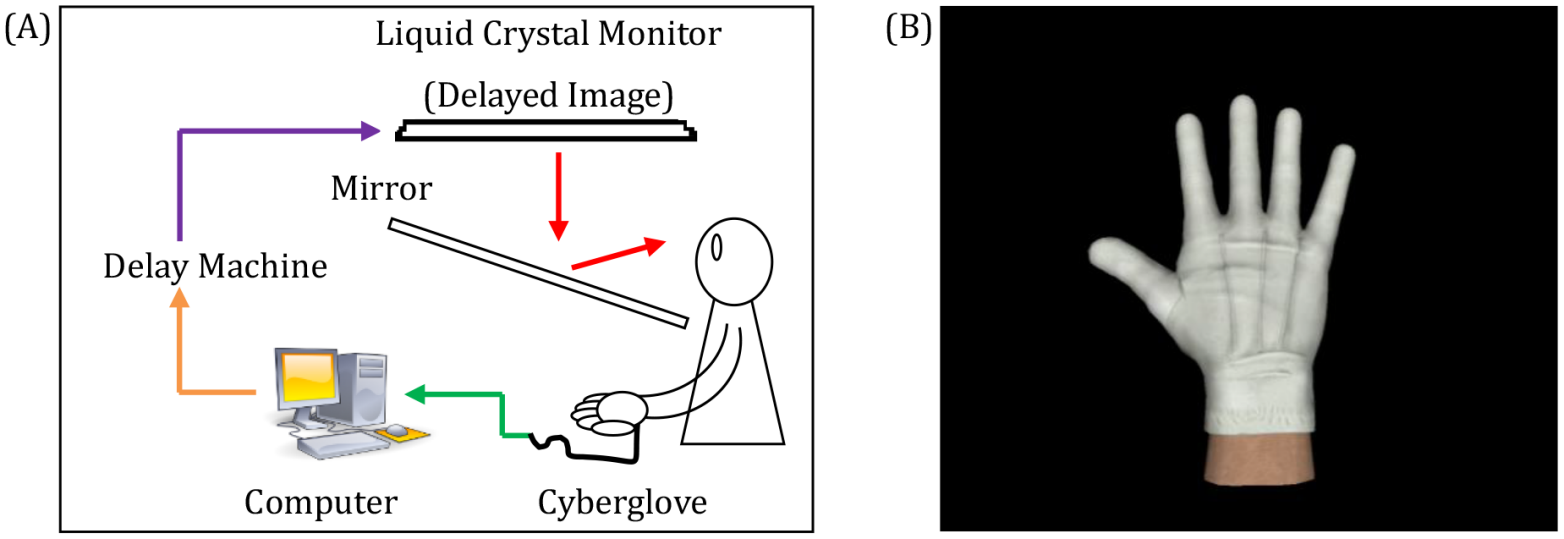
Experimental setup (A) and the virtual hand shown on the liquid crystal display (LCD) (B). The participants observed a delayed virtual image of their hand movement that was recorded using the Cyberglove system. The length of the visual feedback delay ranged from 90 ms to 590 ms at 100-ms intervals.

The participants were asked to sit at a table with their right palm facing down. A mirror was installed above the table, and thus the participants were not able to see their own right hand directly (Fig 1(A)). Participants were instructed to move their hand while using the Cyberglove system (Cyberglove, CyberGlove Systems LLC, San Jose, California) to record their hand movement, which was then transferred to the computer. Recorded data were used to generate the movement of a virtual hand (Fig 1(B)). The participant could see an image of a virtual hand that was presented on a liquid-crystal display monitor (LMD-232W, SONY, Tokyo, Japan) through the reflection of the mirror. The participant could observe the virtual hand as if it was placed on the table roughly at the same position as their own hand by adjusting the angle of the mirror, although the virtual and actual hands were not visually co-located in a strict sense. A hardware device (EDS3305, Eletex, Osaka, Japan) connected to the monitor was used to construct the visual feedback delay. An experiment with six delay conditions ranging from 0 to 500 at 100-ms intervals was conducted. The internal visual feedback delay in the experimental setting measured by a high-speed camera (EX-F1, Casio, Tokyo, Japan) was approximately 90 ms. Therefore, the effective range of the delay conditions was 90–590 ms in this study.

### Procedure

Six delay conditions each for two types of movement (hand opening and closing movement, and free hand movement) were tested for each participant. The participants moved their hand freely according to their own will, including finger movements and wrist flexion. Although there was no rotation of the wrist, the recorded data have 18 degrees of freedom (finger joint angles and wrist flexion), which were fully transferred to the virtual hand. Each run consisted of a 2-min movement period followed by the RoHI questionnaire. The order of the delay conditions was pseudo-random and was counterbalanced across participants.

Participants were exposed to 2 min of stimulation for each delay condition and were then asked to complete a 16-statement questionnaire, which was similar to that used in a previous study [13]. The questionnaires prepared were both in English (original version) and Japanese (translated). For every question, a 7-point Likert scale ranging from −3 (totally disagree) to +3 (totally agree), with 0 indicating that neither agreement nor disagreement (uncertain) was used. The questionnaires resulted in four statements ascribed to the feeling of ownership (e.g., “I felt as if I was looking at my own hand”), and four statements described sensations associated with agency (e.g., “I felt as if I was causing the movement I saw”). The remaining eight statements were control statements, with four describing the ownership sense and the other four describing the agency sense (e.g., “I felt as if I had more than one right hand” and “It seemed as if the image of the hand had a will of its own”). The average score of questionnaire items for each sense was calculated and submitted to further statistical analysis.

To analyze the effect of the length of visual feedback delay on the senses of ownership and agency, one-way repeated measures analyses of variance (ANOVA) were applied separately to the ownership and agency scores. The Mauchly sphericity test was applied, and if significant, the Greenhouse–Geisser correction was adopted. T-tests were used to examine whether there was a significant RoHI effect (higher than zero) with the False Discovery Rate (FDR) control for multiple comparison adjustment. The significance level for all statistical tests was set to 0.05.

## Results

**Fig 2:**
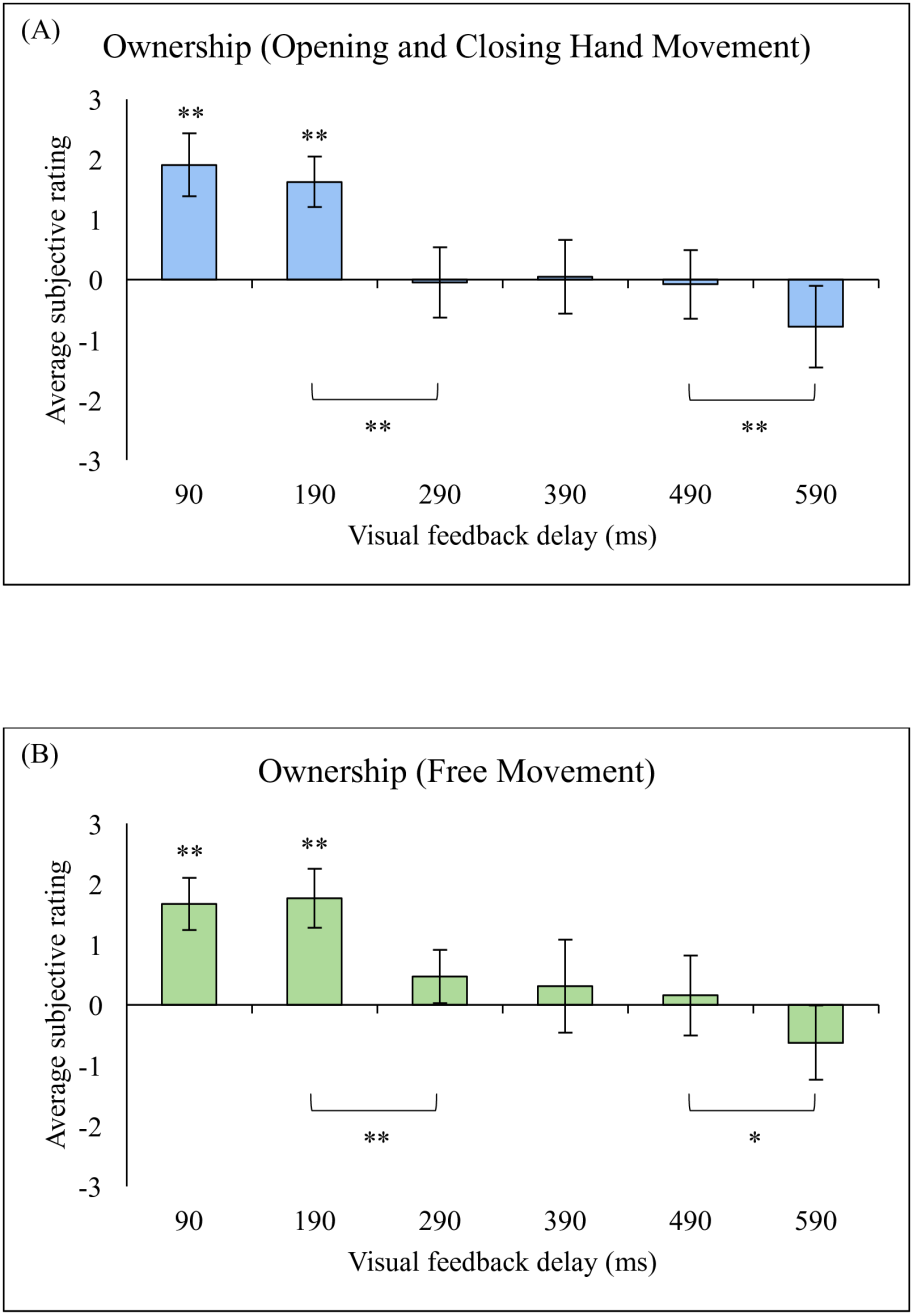
Average subjective rating on ownership sense related questions. (A) Opening and closing hand movement, (B) Free movement. (**p < 0.01, *p < 0.05).

**Fig 3:**
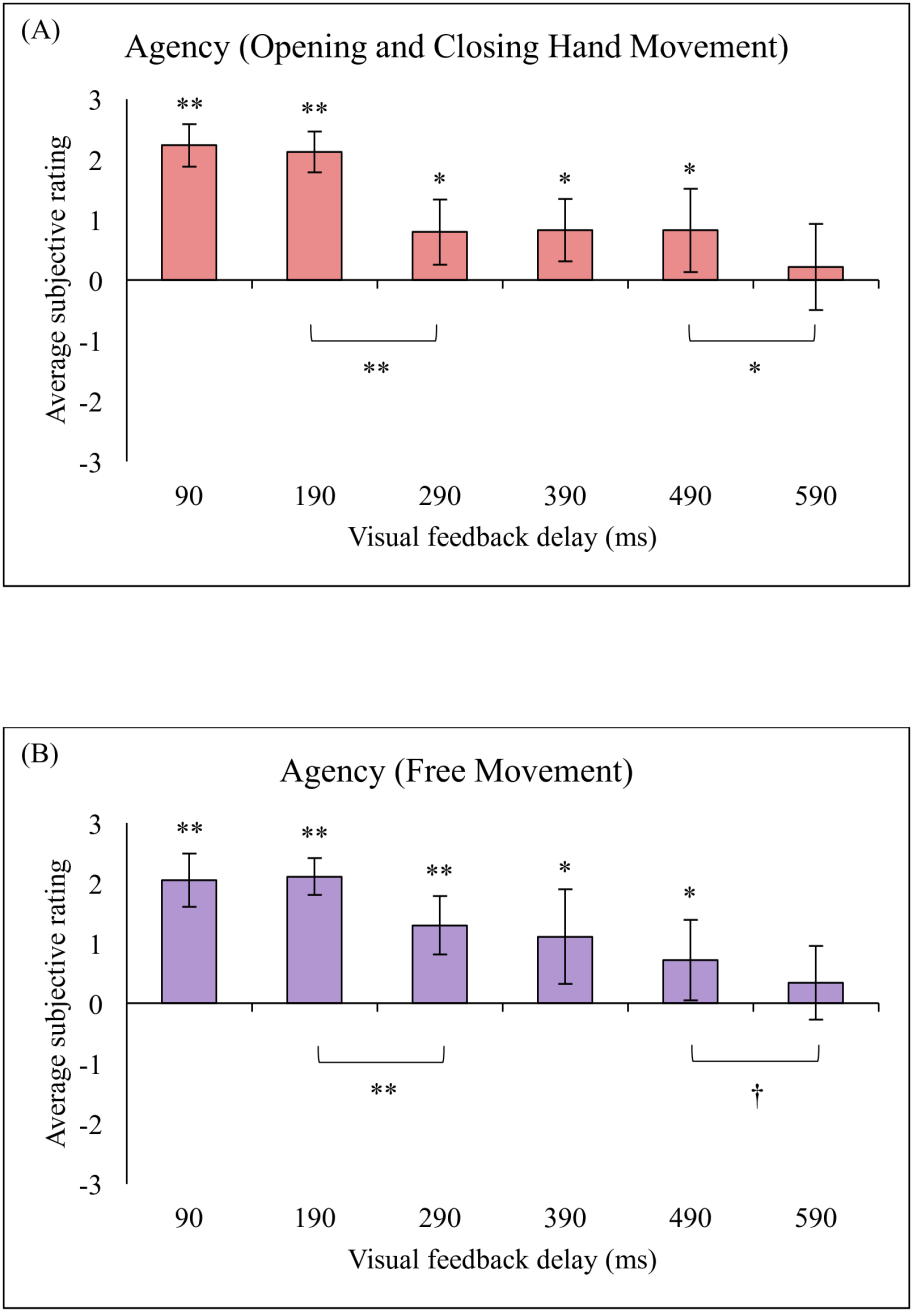
Average subjective rating on agency sense related questions. (A) Opening and closing hand movement, (B) Free movement. (**p < 0.01, *p < 0.05, †p < 0.1).

The averages for the questionnaire items related to the ownership sense (item 1–4) and those related to the agency sense (item 9–12) for all participants in both types of movement (i.e., hand opening and closing movement and free hand movement) are shown in Figs 2 and 3. The results showed that participants rated the 90-ms and 190-ms visual feedback delay conditions above 0 (neutral) for both the ownership sense and the agency sense related questionnaire items (P < 0.01, t-test, FDR-corrected). This result confirms the subjective experience of ownership and agency over the virtual hand. However, participants also rated the 290-ms to 490-ms visual feedback delay conditions above 0 for the agency sense only (P < 0.05, FDR-corrected). In the higher (590-ms) visual feedback delay condition, participants rated the ownership sense below 0 and the agency sense near 0 (P > 0.1). This result confirms that participants did not experience the ownership sense and the agency sense toward the virtual hand in the 590-ms visual feedback delay condition.

A one-way (delay) repeated-measure ANOVA was applied to the ownership sense and agency sense related questions. For the ownership sense, the results showed that there was a significant main effect of delay (F(2.59, 38.87) = 34.97, Greenhouse–Geisser corrected, P < 0.001, for hand opening and closing; F(5, 75) = 20.23, P < 0.001, for free movement). We further applied a post-hoc t-test with multiple comparison adjustment for subsequent analyses and found significant differences between the 190 ms and 290 ms condition pair (t(15) = 7.62, P < 0.01, FDR-corrected, for hand opening and closing; t(15) = 5.69, P < 0.01, FDR-corrected, for free movement) between the 490 ms and 590 ms condition pair (t(15) = 3.96,P < 0.01, FDR-corrected, for hand opening and closing; t(15) = 3.21, P < 0.05, FDR-corrected, for free movement) where there was a sudden decrease in the ownership sense (see Fig 2).

For the agency sense, the results showed that there was a significant main effect of delay (F(2.64, 39.57) = 15.74, Greenhouse–Geisser corrected, P < 0.01, for hand opening and closing; F(2.53, 38.00) = 12.83, Greenhouse–Geisser corrected, P < 0.01, for free movement). We further applied a post-hoc t-test with multiple comparison adjustment for subsequent analyses and found significant differences between the 190 ms and 290 ms condition pair (t(15) = 4.97, P < 0.01, FDR-corrected, for hand opening and closing; t(15) = 4.02, P < 0.01, FDR-corrected, for free movement) between the 490 ms and 590 ms condition pair (t(15) = 3.63, P < 0.05, FDR-corrected, for hand opening and closing; t(15) = 1.93, P < 0.1, FDR-corrected, for free movement) where there was a sudden decrease in the ownership sense (see Fig 3).

**Fig 4:**
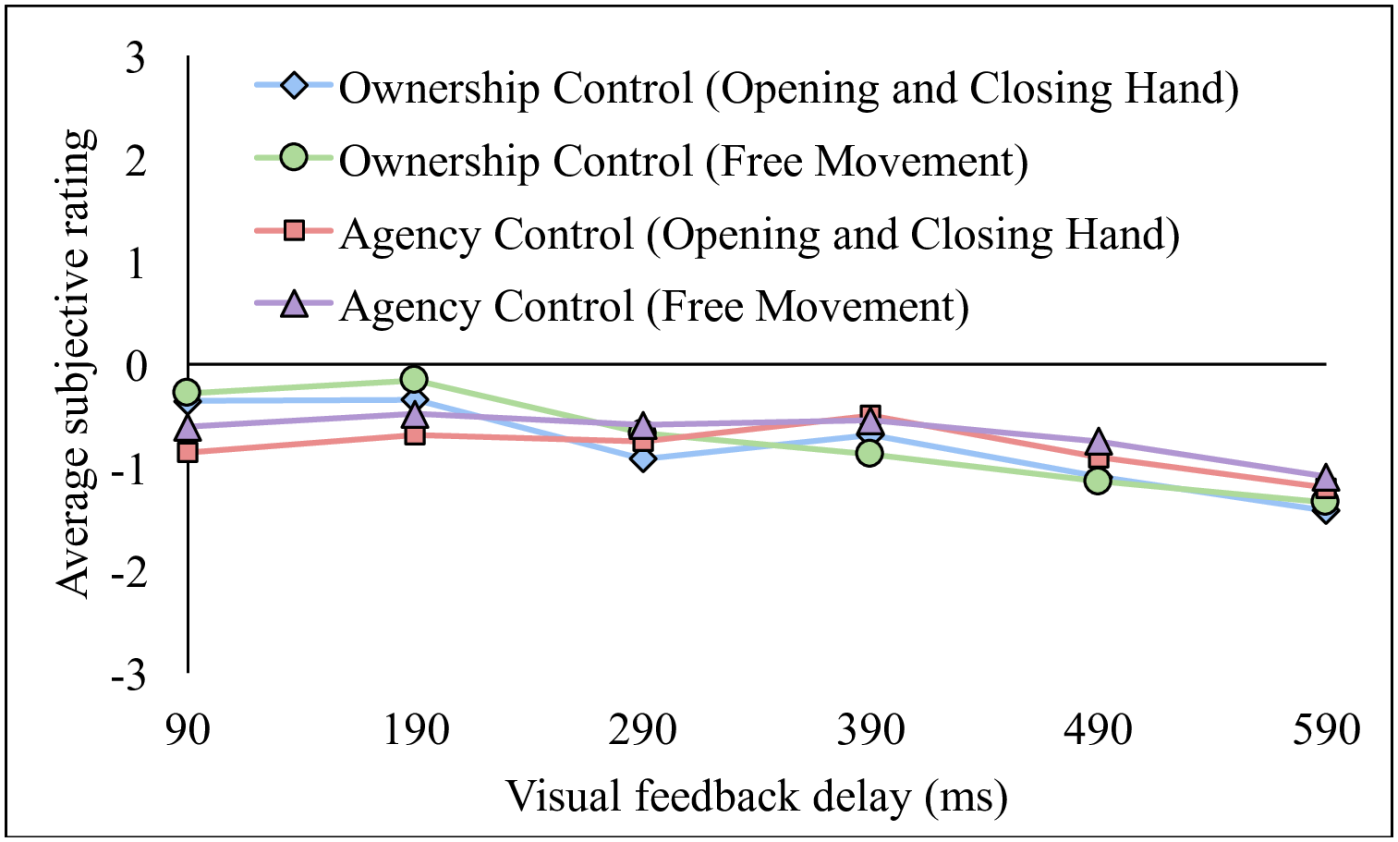
Average subjective rating of the ownership control and agency control related questions.

A one-way (delay) repeated-measure ANOVA was applied separately to the ownership and agency sense control related questions. The results showed that there was no significant main effect of delay among conditions in agency sense control (F(5, 75) = 1.69, P > 0.1, for hand opening and closing; F(5, 75) = 1.99, P > 0.05, for free movement; see Fig 4). However, the results showed that there was a significant main effect of delay among conditions in ownership sense control (F(5, 75) = 8.96, P < 0.01, for hand opening and closing; F(5, 75) = 9.96, P < 0.01, for free movement). We further applied a post-hoc t-test with multiple comparison adjustment for subsequent analyses and found that a significant difference only between the 190 ms and 290 ms condition pair (t(15) = 4.09, P < 0.01, FDR-corrected, for hand opening and closing; t(15) = 4.66, P < 0.01, FDR-corrected, for free movement).

## Discussion

In this study, we focused on the RoHI to investigate the sense of agency and the sense of ownership at the same time. The RoHI is the participant’s illusion of the self-ownership and the self-agency of a virtual (robot) hand that moves consistently with their own hand, and the feeling as if the virtual hand belongs to them. Our result showed that the participants felt a significantly greater effect of the RoHI with a visual feedback delay of less than about 200 ms compared with longer visual feedback delays in both the sense of ownership and sense of agency.

The notion that the duration of time window for multisensory integration in self-body processing is about 200 ms is increasingly supported by recent literature. Some studies have shown that participants can adapt to visuomotor delays when the participant’s action is followed by its visual feedback with a delay of less than 200 ms [14–17]. Other studies have also shown that humans can adapt to auditory or tactile feedback delays within several hundred milliseconds as well [18–20]. For example, Heron et al. showed that exposure to delays of less than 200 ms between a motor action and its associated visual, auditory or tactile sensory feedback leads to vigorous perceptual recalibration of perceptual sensorimotor simultaneity [18]. A recent ERP study has shown the neural basis of the time window for motor-auditory integration using an oddball delayed auditory feedback paradigm [21]. These findings support our notion that a time window of less than about 200 ms is critical for self-body multi-sensory integration.

By referring to the results of the present study, there appear to be three stages of self-body recognition. First, the RoHI occurs in the 90-ms and 190-ms visual feedback delay conditions for both the ownership sense and the agency sense. Second, the agency sense significantly occurs not only for 90–190-ms delays, but also weakly for 290–490-ms delays. However, there was no RoHI effect for the ownership sense in the 290–490-ms conditions. Third, at a delay of 590 ms, no RoHI effect occurred in either the sense of agency or the sense of ownership. These results may offer an important clue to understanding the relationship between the senses of ownership and agency.

The sense of agency showed some differences compared with the sense of ownership in our current results. Our results showed that the participants felt a sense of agency, but not a sense of ownership, even with a 490-ms visual feedback delay. A similarly significant sense of agency in asynchronous and synchronous conditions was also observed in a previous study [22]. Like our result, the participants in that study felt a sense of agency toward the virtual hand image in the asynchronous (490-ms delayed visual feedback) condition. The sense of agency is related to the participants’ sense of controlling the virtual hand. Indeed, the participants could control the virtual hand, even though it was delayed from their own hand movement, in the 290–490-ms delay conditions, which, however, is significantly different when compared with the 90–190-ms conditions.

There are various explanations concerning the relationship between the ownership sense and the agency sense. According to a previous study, different cerebral activity occurs for the sense of agency compared with the sense of ownership, which suggests two different and independent systems [15]. The independent model assumes that the ownership sense and the agency sense are two separate components or processes. In fact, an analysis of the questionnaires in the present study shows that the ownership sense and the agency sense demonstrate different properties. Conversely, the additive model postulates the interrelationship between the agency and the ownership senses. Caspar et al. [11] examined the sense of agency and the sense of ownership using a robotic hand under simple synchronous and asynchronous conditions. Their analysis of the questionnaires revealed that both the agency and the ownership senses were related, supporting the additive model. Our results observed in the baseline (90-ms) conditions offer support for the additive model, with a positive correlation between the agency sense and the ownership sense scores on the questionnaire (r = 0.68, P < 0.01). Because the agency sense is more susceptible than the ownership sense in our experiment, we consider that the agency sense could be a prerequisite for the ownership sense. However, it is obvious that we need further methodological advancement in future studies to solve these problems.

Our result showed that there was a significant main effect of delay among conditions on the ownership sense control for both opening and closing hand movement and free movement. This result is somewhat unexpected, but this tendency that scores for ownership sense control becomes lower in the asynchronous than in the synchronous condition was observed in previous studies (Kalckert and Ehrsson, 2012; 2014), although it was not mentioned explicitly in the literature. This may imply that the ownership sense control is susceptible to conditional manipulation in RoHI experiments. Nevertheless, because we did not find any significant first-level effect in the ownership sense control in each delay condition, as well as in the agency sense control, we consider that the conditional difference in the ownership sense control has little effect on our interpretation of the RoHI effects in our experiment.

In conclusion, temporal multisensory integration is important for the production of the self-body senses, namely the sense of ownership and the sense of agency. Our study suggests that visual feedback delays should be less than about 200 ms to produce the strong self-body sense by integrating vision and movement.
